# Who Watches the Watchmen: Roles of RNA Modifications in the RNA Interference Pathway

**DOI:** 10.1371/journal.pgen.1006139

**Published:** 2016-07-21

**Authors:** Samantha B. Shelton, Calder Reinsborough, Blerta Xhemalce

**Affiliations:** Department of Molecular Biosciences, University of Texas at Austin, Austin, Texas, United States of America; University of Cambridge, UNITED KINGDOM

## Abstract

RNA levels are widely thought to be predictive of RNA function. However, the existence of more than a hundred chemically distinct modifications of RNA alone is a major indication that these moieties may impart distinct functions to subgroups of RNA molecules that share a primary sequence but display distinct RNA “epigenetic” marks. RNAs can be modified on many sites, including 5′ and 3′ ends, the sugar phosphate backbone, or internal bases, which collectively provide many opportunities for posttranscriptional regulation through a variety of mechanisms. Here, we will focus on how modifications on messenger and microRNAs may affect the process of RNA interference in mammalian cells. We believe that taking RNA modifications into account will not only advance our understanding of this crucial pathway in disease and cancer but will also open the path to exploiting the enzymes that “write” and “erase” them as targets for therapeutic drug development.

## Introduction

**Fig 1:**
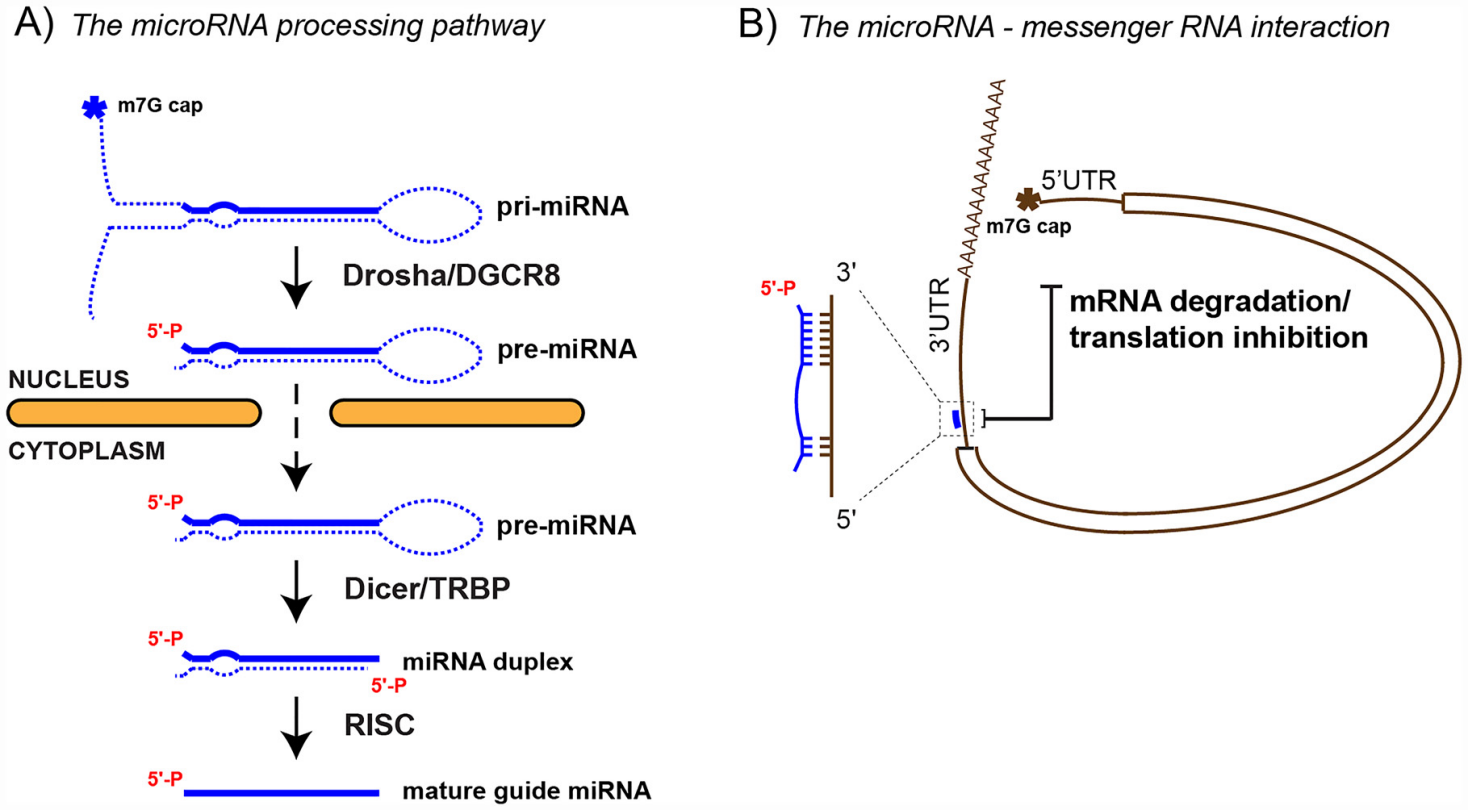
Simplified schematic of (A) the miRNA biogenesis pathway and (B) the interaction between the mRNA and miRNA. **(A)** The primary miRNA precursor (pri-miRNA) is synthesized by RNAP II. The pri-miRNA is first cleaved by Drosha to release a hairpin loop–shaped RNA called pre-miRNA. The loop of this pre-miRNA is further cleaved by Dicer to generate a miRNA duplex. The miRNA duplex is dissociated and the passenger strand (dashed line) is discarded while the guide strand is loaded onto the Argonaute protein to form an active RISC complex. **(B)** Example of miRNA–target mRNA interaction by base-pairing mainly at the seed region of miRNA (nt 2–8) but also on other downstream regions of the miRNA. In the mRNA, the coding region is represented as a double line. **N.B.** The asterisks indicate the m7G cap (7-methyl-guanosine with 5′, 5′-triphosphate linkage).

In higher multicellular eukaryotes, a crucial gene regulatory step takes place posttranscriptionally through RNA interference using microRNAs (miRNA). miRNAs are 18–24 nt, short, noncoding RNAs that target the RNA interference (RNAi) effector complex RNA-induced silencing complex (RISC) to specific messenger RNAs (mRNAs) through partial base pairing to sequences predominantly found in their 3′ untranslated regions (3′UTR) (Fig 1) [1]. This mRNA–miRNA interaction can result in either decreased mRNA stability and/or inhibition of translation into proteins, resulting in reduced protein expression (Fig 1). Canonical miRNAs are themselves synthesized from larger RNA Polymerase II (RNAP II) transcripts and undergo extensive processing until they reach the mature single-stranded form that is loaded into RISC (Fig 1A). Based on sequence, every miRNA has many predicted target mRNAs, and conversely, 40%–50% of mRNAs have one or more predicted miRNA target sites (Fig 1B) [2]. However, the rules that govern RNAi targeting are not well understood, making it difficult to predict whether a putative mRNA–miRNA pair will result in an RNA interference event in a given cell [3]. This could partially result from target competition, determined by the relative expression of mRNAs with the miRNA targeting site or other noncoding RNAs acting as miRNA sponges [4]. However, it is also possible that posttranscriptional events, such as RNA modifications, regulate specific mRNA–miRNA interactions. It is conceptually easy to envision how a genetic mutation in DNA that is copied into the mRNA can lead to a loss of regulation by a miRNA. An elegant example of this is a single nucleotide polymorphism (SNP) in the 3′UTR of KRAS that alters let-7 miRNA binding and leads to a higher incidence of non–small cell lung cancer [5]. By extension, “epigenetic” modifications of RNAs could assume this role by not only regulating the levels of messenger or miRNAs but also by masking or enhancing miRNA binding sites. In this review, we will first provide a brief overview of different types of RNA modifications—including those to 5′ and 3′ RNA ends as well as internal base modifications such as Adenine N6 and Cytosine C5 methylations—and RNA editing. These RNA modifications will then be discussed based on the type of functional RNAs—messenger or miRNAs—that they occur on to emphasize that miRNA modifications can affect both miRNA processing and mRNA–miRNA interactions. We will further touch upon the challenges of detecting RNA modifications, especially on small RNAs, highlighting recent technological advances that could be exploited to determine the miRNA modification landscape. Finally, we will pinpoint the consequences of the rising field of epitranscriptomics for our understanding of myriad diseases, including cancer, and how RNA modifications and the enzymes that write and erase them could be exploited for diagnostic markers and drug development.

## RNA Modifications

**Fig 2:**
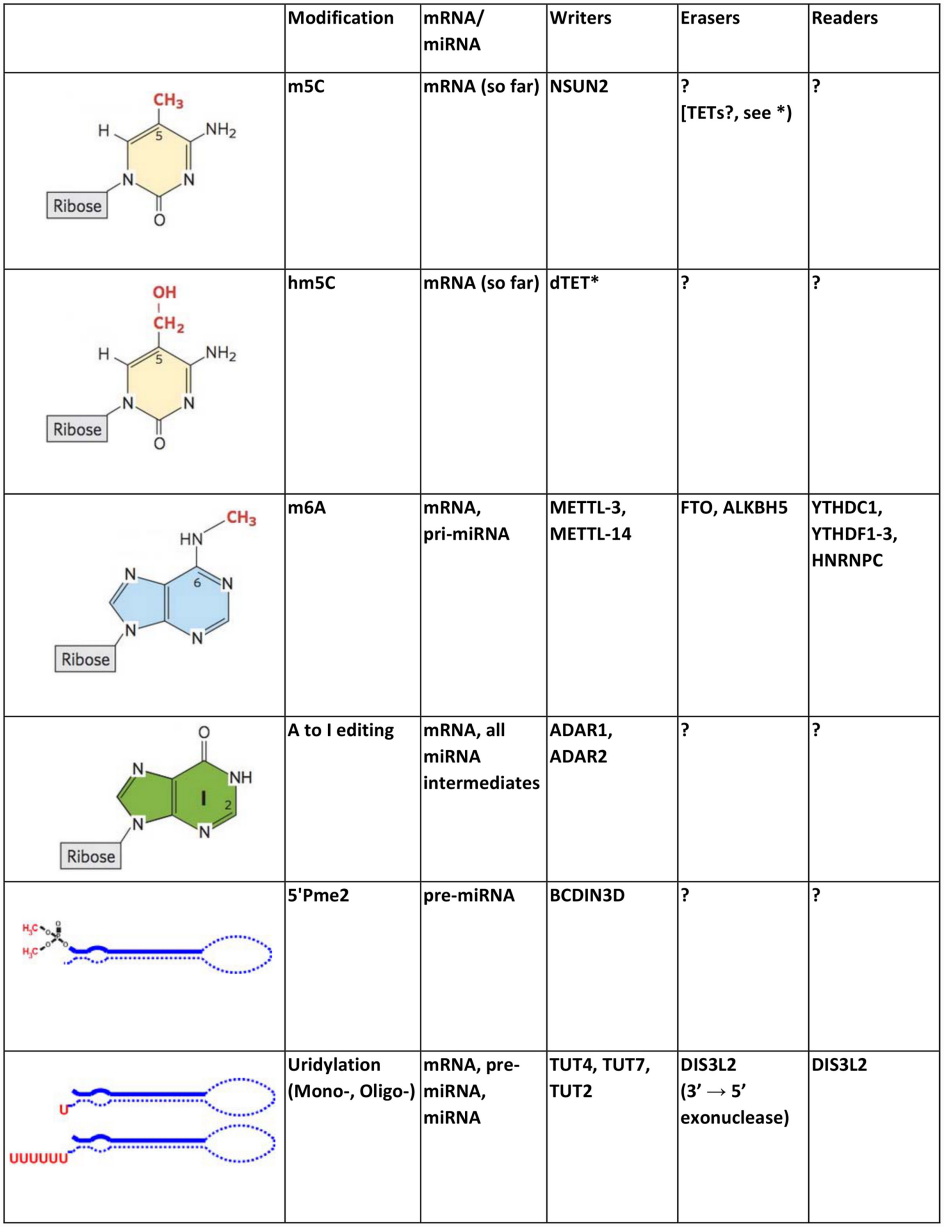
List of known messenger or miRNA modifications, with chemical structures, abbreviation, writers, erasers, and readers. *The *Drosophila* dTET enzyme has recently been shown to hydroxylate 5meC on RNA [11].

There are more than a hundred chemically distinct RNA modifications [6] (http://mods.rna.albany.edu) that can be divided into several subcategories depending on their chemical structure, the target site on the RNA polymer, and the functional type of RNA. Here, we will focus on the modifications that have been detected in the two RNAs directly relevant to RNA interference: mRNAs and miRNAs. As in other RNAs, mRNA and miRNA modifications can be added to their 5′ and 3′ ends or internally to specific bases. During transcription by RNAP II, both mRNAs and pri-miRNAs receive a 7-methylguanosine cap (m7G) on their 5′ end and a poly-A tail on their 3′ end (Fig 1) [1]. For more details on these RNA modifications, we refer you to this excellent review by Bentley [7]. Other end modifications specific to miRNA intermediates are 5′ phosphomethylation [8] and the untemplated addition of one [9] or more [10] uridylyl residues on the 3′ end (Fig 2). The most prevalent internal modifications are adenosine to inosine (A to I) editing and methylations on the carbon 5 of the pyrimidine ring on cytosines, m5C (essentially the same modification catalyzed by the bona fide epigenetic DNA methyltransferases), and the nitrogen on carbon 6 of the purine ring on adenosines, m6A (Fig 2).

### RNA end modifications

Apart from the cotranscriptional m7G capping and polyadenylation of mRNAs and pri-miRNAs, two end modifications, 5′ phosphomethylation (5′Pme2) and 3′ uridylation, have been shown to regulate the biogenesis and stability of specific miRNAs in mammalian cells.

#### 5′ phosphomethylation

**Fig 3:**
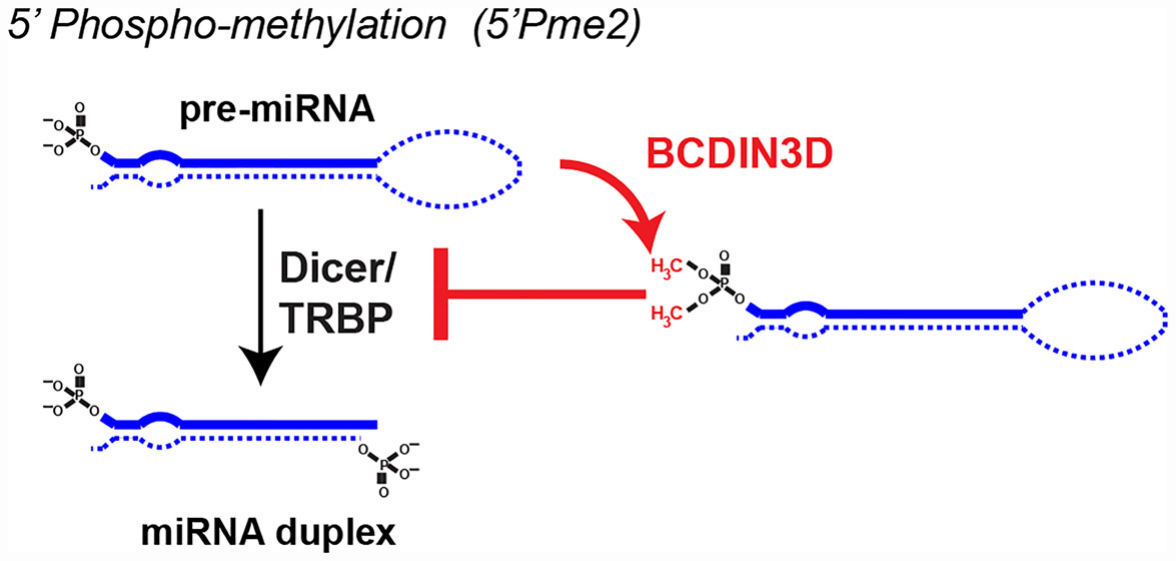
Model for the mode of action of the BCDIN3D RNA methyltransferase on the biogenesis of specific miRNAs. BCDIN3D’s enzymatic activity consists in the methylation of the two available oxygen moieties of the 5′ monophosphate, which removes the 5′ monophosphate charge and makes it bulkier. This methylation blocks the processing of specific miRNAs [8], possibly through perturbing the interaction of the 5′ monophosphate with its binding pocket in Dicer [8,14].

A key feature of the miRNA biogenesis pathway is the generation of 5′ mono-phosphate (5′P) ends by Drosha and Dicer (Fig 1). The 5′P is bound by specific, positively charged pockets within Dicer and Ago2 to ensure efficient and accurate pre-miRNA processing as well as miRNA–RISC stability [12–15]. The BCDIN3D RNA methyltransferase methylates both available oxygen moieties of the 5′P on specific pre-miRNAs, including pre-miR-145 (Figs 2 and 3) [8]. This methylation abolishes the negative charge of the 5′P and makes the 5′ end bulkier, resulting in reduced processing of pre-miRNAs by Dicer [8]. It is also important to note that 5′ ends are commonly modified on commercially available small interfering RNAs (siRNA) to stimulate Ago2/RISC loading. For example, in the ON-TARGETplus siRNA duplexes from Dharmacon, only the antisense oligonugleotide is 5′ monophosphorylated in order to limit off-target effects by minimizing the loading of the unphosphorylated sense siRNA onto Ago2/RISC. These examples highlight the importance of the 5′P end in the RNAi pathway and suggest several potential levels of regulation involving the 5′ end.

#### 3′ uridylation

Uridylation consists in the untemplated addition of one or more uridilyl residues on the RNA 3′ end. Uridylation of let-7 family pre-miRNAs by terminal uridylyl transferase enzymes (TUTases) constitutes a beautiful example of how the degree of modification is critical for determining the fate of the modified miRNA precursors. Indeed, the preferred substrate of Dicer is a pre-miRNA with a 2 nt 3′ overhang [9]. Thus, on class II let-7 pre-miRNAs, which have a 1 nt 3′ overhang, the addition of one uridine or monouridylation by TUT7/4/2 restores a 2 nt 3′ overhang and stimulates Dicer processing [9]. However, oligouridylation of pre-miRNAs by TUT7/4 has the opposite effect, preventing their processing and leading to their degradation [10,16]. Oligouridylation can be stimulated by at least two different processes: either by the reprogramming factor and oncogenic driver LIN28 [10,16] or, in the absence of LIN28, by abnormal 5′ overhangs or trimmed 3′ ends [17]. Interestingly, DIS3L2 has the dual ability to recognize the oligo-U tail [18] and to degrade the oligouridylated pre-miRNA through its 3′ → 5′ exonuclease activity [19].

### RNA base modifications

Base modifications constitute the most diverse body of known RNA modifications [6]. They have mostly been analyzed in tRNAs, which have the advantage of being abundant and thus amenable to biochemical purification and analysis [20]. However, the development of new methods using next-generation sequencing on modified RNA enriched through specific affinity purification methods has allowed the analysis of a set of modifications on the less abundant RNAs, including mRNAs.

#### A to I editing

A to I editing corresponds to the conversion of adenosine to inosine by removal of the amino group at carbon 6 of the adenine ring (Fig 2). On mRNAs and miRNAs, A to I editing is catalyzed by the ADAR1/2 (adenosine deaminase acting on RNA) proteins (Fig 2) (extensively reviewed in [21]). Inosine changes the base pairing properties of the edited adenosine, as the inosine at the wobble nucleotide in a tRNA anticodon can form two hydrogen bonds with either C, U, or A bases, while inosine in mRNA is read as a G base during translation. Consequently, A to I editing in mRNAs leads to specific changes in protein sequence [22] but may also alter miRNA binding sites in their 3′UTRs [23]. However, miRNA–mRNA interactions are most commonly altered by A to I editing in the seed sequence of the guide strand of the miRNA [24]. Additionally, by affecting base paring, A to I editing perturbs double-stranded structures in pri- and pre-miRNAs that alter Drosha and Dicer processing of these precursors [24,25]. Thus, A to I editing provides a clear example of how an RNA modification affects RNAi by perturbing either mRNA–miRNA base pairing or RNA secondary structures.

#### Adenosine 6 methylation (m6A)

**Fig 4:**
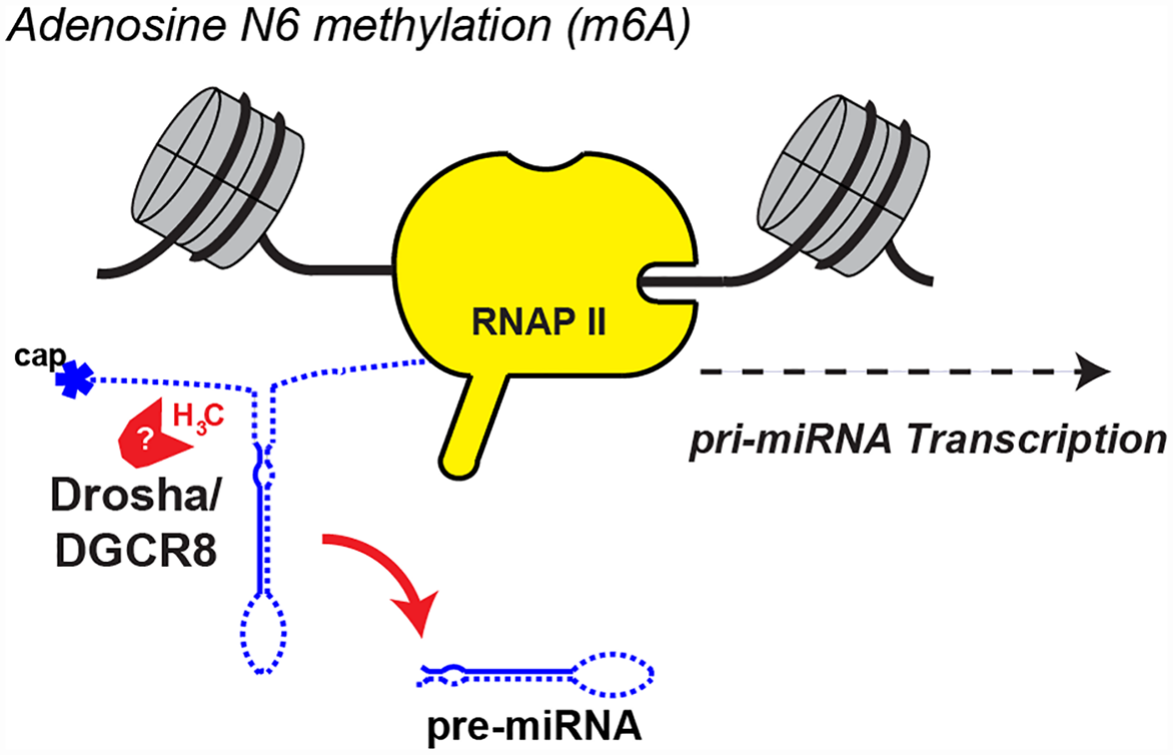
Model for how m6A may stimulate pri-miRNA processing. m6A is deposited on pri-miRNA by METTL3 and is thought to stimulate the recruitment of Drosha/DGCR8 for co-transcriptional processing of pri-miRNA to pre-miRNA [27]. The question mark is to highlight that the identity of the full set of m6A readers in pri-miRNAs is unknown. In yellow is shown RNAP II on DNA surrounded by nucleosomes.

m6A is the most abundant base modification in mRNA [26] but has also recently been found on pri-miRNAs [27]. The m6A mark regained widespread interest more than 30 years after its discovery on mRNA when He and coworkers discovered that the fat- and obesity-associated protein FTO is a m6A demethylase [28]. m6A is deposited on mRNA by a complex comprising the METTL3-METTL14 methyltransferases and WTAP [29–31] and removed by the FTO and ALKBH5 jumonji demethylases [28,32]. RNA immunoprecipitation with anti-m6A antibodies coupled to next-generation sequencing (m6A-RIP-seq) found this modification enriched in the proximity of stop codons and on 3′UTRs [33,34] (see “Hopes and Challenges in the Rising RNA Modification Field” for an overview of transcriptome-wide methods). Another related method using cross-linking of the anti-m6A antibody to RNA (m6A-CLIP-seq) found that m6A is in fact enriched 150–400nt after the start of the last exon, which coincides with the stop codon on most, but not all, mRNAs [35]. Both m6A-RIP-seq and m6A-CLIP-seq methods showed significant m6A enrichment on 3′UTRs [34,35], suggesting that m6A could regulate mRNA function through processes that operate via the 3′UTR, such as RNAi via miRNAs or alternative polyadenylation (APA). Meyer et al. found that two-thirds of 3′UTRs that contain a m6A modification also contain a predicted miRNA binding site [34]. Moreover, Ke et al. identified a significant overlap of m6A with Ago binding sites in 3′UTRs of mRNAs purified from mouse brains but not for other 3′UTR binding proteins [35]. These correlations suggest a possible connection between m6A methylation of mRNAs and RNAi. In addition, Ke et al. investigated the effect of knocking down the m6A methylase complex on APA, finding that about one-sixth of examined genes changed their predominant APA with both types of switches, distal to proximal (63%) or proximal to distal (37%) [35], upon m6A methylase depletion. Given that longer 3′UTRs are more likely to be regulated by miRNAs [36], APA could be another way for m6A to control RNAi. Importantly, Alarcon et al. found by both m6A-RIP-seq and METTL3 CLIP-seq that METTL3 targets primary miRNAs for m6A methylation [27]. Moreover, DGCR8 interacts with m6A methylated RNA, and m6A methylation stimulates pri-miRNA processing in vitro [27]. Consistent with this, METTL3 knockdown led to a global downregulation of mature miRNAs [27]. Together, these results suggest that m6A directly marks primary miRNAs for processing by Drosha/DGCR8 (Fig 4). The m6A mark has been shown to increase the binding of specific “reader” proteins, including YTHDC1, YTHDF1–3, and HNRNPC (Fig 2) [33,37–39]; however, the identity of the full set of proteins that mediate binding of Drosha/DGCR8 to m6A methylated pri-miRNAs remains to be determined [27,40]. Finally, it is interesting to note that m6A strongly inhibits the activity of ADAR2 in vitro, suggesting yet another possible mode of action for m6A [41].

#### Cytosine 5 methylation (m5C) and 5 hydroxymethylation (hm5C)

m5C is the second-most abundant base modification in mRNA, around 20 times less abundant than m6A [26]. This modification has been well studied in tRNAs where DNMT2 and NSUN2 m5C methylate specific residues located on or around the anticodon and/or the variable loop of specific tRNAs to protect them from cleavage (reviewed in [20]). Interestingly, a study that used genome-wide bisulfite sequencing (see next section) showed that NSUN2 may also methylate mRNAs [42]. In addition, m5C showed a slight overrepresentation on 5′ and 3′UTR of mRNAs; however, the significance of these findings for mRNA function remains obscure. Interestingly, hm5C has recently been detected in RNA [43]. In DNA, hm5C by the Tet dioxygenase enzymes is thought to be the first step towards demethylation [44], raising the intriguing possibility that m5C is a reversible mark on RNA too (Fig 2).

## Hopes and Challenges in the Rising RNA Modification Field

The recent discovery of new writers, erasers, and readers of RNA modifications with important cellular phenotypes has triggered the rebirth of the RNA modification field [45]. Despite their pervasiveness, the function and the molecular mechanism of action of many RNA modifications, including m6A, remain obscure. Moving forward, the establishment of rigorous and sensitive methodologies will be necessary to fill this important gap in our knowledge. A key step toward achieving this goal is the ability to identify modified RNAs as well as to map base modifications at the nucleotide resolution level. RNA modifications that change the base-pairing properties or RNA sequence, such as editing and uridylation, can already be detected with nucleotide resolution. However, other base modifications require a conversion or antibody-mediated enrichment step. For example, in the case of m5C [42], sodium bisulfite treatment leads to deamination of unmethylated cytosines to uracil, but leaves m5C cytosines intact. The resulting uracils are amplified in the subsequent reverse transcription and PCR steps as thymines, whereas m5Cs are amplified as cytosines. Sequence comparison between mock and sodium bisulfite-treated RNAs then allows for determination of the position(s) and the ratio of m5C. A similar method does not currently exist for m6A. The most commonly used method is m6A-RIP-seq, in which m6A methylated RNAs are enriched with an anti-m6A antibody prior to next-generation sequencing [33,34]. m6A-RIP-seq is effective in identifying approximate m6A locations, but because the mRNA is sheared into ~100 nucleotide fragments that often contain multiple putative m6A sites, it is not possible to resolve m6A at the nucleotide level. A more recent method called m6A-CLIP/IP adds a step that uses ultraviolet (UV) light to crosslink the anti-m6A antibody to the m6A-modified RNA fragment [35]. Upon antibody digestion with proteinase K and sequencing, nucleotide insertions are observed adjacent to verified m6A sites [35]. Interestingly, Ke et al. also observed frequent truncations at m6A sites with both RIP and CLIP methods, suggesting that m6A may cause the reverse transcriptase to fall off during cDNA synthesis [35]. These observations reveal a potential for biases introduced by commonly used molecular biology enzymes when dealing with modified RNAs. On a positive note, these biases could also be exploited to identify modification sites on RNA. For example, it could be of interest to engineer reverse transcriptases that can incorporate another nucleotide than thymine when encountering a m6A-modified adenosine during cDNA synthesis. Such designer reverse transcriptases could in principle be adapted to any RNA modification.

The challenges mentioned above are applicable to all modified RNAs. However, another challenge specific to the miRNA pathway is the lack of reliable methods to amplify and sequence precursor miRNAs. While this may be a consequence of their supposed short half-life as intermediates, it is also possible that it is the combination of their stable hairpin structure and the presence of RNA modifications that makes them poor substrates for cDNA synthesis and thus deep sequencing analysis. A similar problem is encountered with highly modified tRNAs, despite their abundance [46]. To resolve this issue, two recent methods used a cocktail of engineered demethylases to remove base methylations that act as roadblocks during cDNA synthesis, resulting in significantly improved coverage and quantitative tRNA sequencing [47,48]. The application of such methods to pre-miRNA sequencing could yield invaluable insights into how these precursors regulate the miRNA pathway.

Accurately detecting RNAs and their modifications may prove to be important not only from a basic scientist’s perspective, but also from a biomarker development point of view. Indeed, the absence or presence of specific RNA modifications in disease setting may be more informative than simply measuring RNA levels. This is very likely given the involvement of the writers, erasers, and readers of RNA modifications in cellular processes highly relevant to human disease [20]. All methods used to date (bisulfite conversion, m6A-RIP-seq, etc.) require large amounts of starting material that are often not available for patient derived samples. Thus, the discovery of sensitive and high-resolution methods to detect RNA modifications may be transformative for biomarker development and use in the clinic.

As highlighted throughout this review, an increasing number of observations point to the important role played by RNA modifications in the miRNA biogenesis pathway and RNA interference. Given the importance of miRNAs in numerous cellular processes and their deregulation in many diseases, including cancer, miRNA inhibitors and miRNA mimics are being considered for therapeutic purposes [49]. While nucleic acid–based miRNA inhibitors or mimics have shown excellent potential in the laboratory setting, their use in the clinic is problematic because their uptake is usually limited to the liver and kidney [49]. For this reason, we believe that the focus should instead be on regulators of the miRNA pathway, such as enzymes, writers, or erasers of RNA modifications [50–52]. Enzymes are more likely to be targeted with small molecule drugs and could possibly affect many miRNAs simultaneously. Thus, taking into account RNA modifications will not only advance our understanding of the RNA interference pathway in disease and cancer but will also open the path to exploiting the enzymes that write and erase them as targets for therapeutic drug development.
